# Implementation of large-scale pooled testing to increase rapid molecular diagnostic test coverage for tuberculosis: a retrospective evaluation

**DOI:** 10.1038/s41598-023-41904-w

**Published:** 2023-09-16

**Authors:** Comfort Vuchas, Pride Teyim, Beh Frankline Dang, Angela Neh, Liliane Keugni, Mercy Che, Pantalius Nji Che, Hamada Beloko, Victor Fondoh, Norah Nyah Ndi, Irene Adeline Goupeyou Wandji, Mercy Fundoh, Henri Manga, Cyrille Mbuli, Jacob Creswell, Annie Bisso, Valerie Donkeng, Melissa Sander

**Affiliations:** 1 Center for Health Promotion and Research, Bamenda, Northwest Cameroon; 2 Tuberculosis Reference Laboratory Douala, Douala, Littoral Cameroon; 3 Bamenda Regional Hospital, Bamenda, Northwest Cameroon; 4 Baptist Convention Health Services and Baptist Institute of Health Sciences, Bamenda, Northwest Cameroon; 5 National TB Program- Littoral Region, Douala, Littoral Cameroon; 6 National TB Program- Northwest Region, Bamenda, Northwest Cameroon; 7 National TB Program, Yaoundé, Center Cameroon; 8 Stop TB Partnership, Geneva, Switzerland; 9 Centre Pasteur du Cameroun, Yaoundé, Center Cameroon

**Keywords:** Diagnosis, Laboratory techniques and procedures, Tuberculosis

## Abstract

In 2021, only 6.4 million of the 10.6 million people with tuberculosis (TB) were diagnosed and treated for the disease. Although the World Health Organization recommends initial diagnostic testing using a rapid sensitive molecular assay, only 38% of people diagnosed with TB benefited from these, due to barriers including the high cost of available assays. Pooled testing has been used as an approach to increase testing efficiency in many resource-constrained situations, such as the COVID-19 pandemic, but it has not yet been widely adopted for TB diagnostic testing. Here we report a retrospective analysis of routine pooled testing of 10,117 sputum specimens using the Xpert MTB/RIF and Xpert MTB/RIF Ultra assays that was performed from July 2020 to February 2022. Pooled testing saved 48% of assays and enabled rapid molecular testing for 4156 additional people as compared to individual testing, with 6.6% of specimens positive for TB. From an in silico analysis, the positive percent agreement of pooled testing in pools of 3 as compared with individual testing for the Xpert MTB/RIF Ultra assay was estimated as 99.4% (95% CI, 96.6% to 100%). These results support the scale-up of pooled testing for efficient TB diagnosis.

## Introduction

Pooled testing is a method used to improve the efficiency of clinical testing when test positivity rates are relatively low^1,2^. In pooled testing, also known as group testing, samples from multiple individuals are combined prior to testing^3,4^. A simple and widely used pooled testing approach is to perform testing in two stages, as described by Dorfman^1^. For any pooled test with a positive result, each individual specimen from the positive pool is then tested individually and the individual result is reported; for any pooled test with a negative result, all individual specimens within that pool are reported as negative. Pooled testing can yield substantial savings in test reagents, personnel time, and equipment usage when the pool size is chosen judiciously based on the positivity rate in the population tested. However, while pooled testing can be used to improve testing efficiency, it can also result in a decrease in analytical sensitivity due to specimen dilution. The trade-off between improved test efficiency and reduced test sensitivity contributes to decisions about whether to use pooled testing in various situations. Pooled testing has been used over several decades for a range of clinical applications, including for disease screening, treatment monitoring, and infectious disease surveillance^5–8^.

Pooled testing may be preferred in resource-constrained situations such as public health emergencies. In 2020, pooled testing was identified as an important strategy during the COVID-19 pandemic^9–14^. Many countries implemented pooled testing for detecting the SARS-CoV-2 virus with polymerase chain reaction (PCR)-based assays, with varying pool size depending on disease prevalence^15–18^. From these reports, pooled testing with various pooling strategies enabled estimated reductions of 40–90% in the number of assays needed as compared to individual testing, with only minor decreases in viral detection. In these routine testing applications, only positive pools were typically retested, so the positive percent agreement of pooled with individual testing for different assays and pool sizes was estimated either with validation studies or with retrospective estimations. Following recommendations of the US Food and Drug Administration, the positive percent agreement can be estimated for pooled testing as compared to individual testing using in silico analyses^19,20^. The in silico analysis is performed with the quantitative measure of the cycle threshold, which is the number of cycles of amplification required for the detection (or crossing of the specified fluorescence threshold) of the PCR amplification target. After obtaining the shift in cycle threshold for specimens that tested positive both in pools and individually for a particular pool size and assay target, this shift is then applied to the distribution of cycle threshold values of an historical clinical data set to estimate the positive percent agreement of pooled as compared to individual testing. These approaches to evaluate testing efficiency and to estimate positive percent agreement of pooled compared to individual testing for detection of the SARS-CoV-2 virus are general and can be extended to tests for other diseases.

Tuberculosis (TB) affects people primarily in resource-limited settings. In 2021 there were 1.6 million deaths due to the disease^21^. Rapid molecular diagnostics facilitate early and accurate detection of people with TB, contributing to improved patient outcomes and reduced disease transmission. The World Health Organization (WHO) recommends the use of sensitive molecular methods, including the Xpert MTB/RIF (Xpert) and the Xpert MTB/RIF Ultra (Ultra) assays (Cepheid, USA), both PCR-based tests, as the initial diagnostic test for TB^22,23^. However, many people are still tested for TB using sputum smear microscopy, which has a sensitivity of only 50 to 60% versus TB culture, as compared to the sensitivity of approximately 85% for the Xpert assay and 91% for the Ultra assay in adults with pulmonary TB^24,25^. The scale-up of rapid molecular testing for TB has been hindered by issues including the relatively high cost of assays, limited laboratory capacity for providing molecular testing, and equipment maintenance issues^26–32^. Globally, only 38% of people newly diagnosed with TB in 2021 received a WHO-recommended rapid molecular test as an initial diagnostic test for TB^21^.

Several research studies have evaluated the efficiency and diagnostic accuracy of pooled testing of sputum samples for TB detection with both the Xpert and Ultra assays^33–38^. In a systematic review of studies where each specimen was tested both individually and in pools, Cuevas et al. reported that the positive percent agreement for pools of four samples as compared with individual samples was 91% for the Xpert MTB/RIF assay and 98% for the Xpert MTB/RIF Ultra assay^39^. Subsequent studies have reported similarly small reductions in testing accuracy with large improvements in testing efficiency and significant cartridge and cost savings when using these assays for pooled testing for TB detection^36,37,40^. However, despite these results that highlight the good potential value of pooled testing to improve the detection of TB, pooled testing has not yet been widely adopted to increase molecular testing coverage for routine TB diagnosis. In the recently published WHO standard on universal access to rapid tuberculosis diagnostics, pooled testing is identified as an innovative approach to assist in meeting this standard^23,41^.

Cameroon has a TB incidence of 164 people per 100,000 and an estimated treatment coverage of only 50% of the people with TB in the country in 2021. WHO reporting shows that only 22% of people notified with TB in Cameroon received a WHO-recommended rapid diagnostic as an initial test for TB^23^. In 2020, due to supply chain disruptions related in part to the COVID-19 pandemic, it became apparent that Cameroon was likely to have a prolonged shortage of Xpert and Ultra testing reagents. In response, several of the TB testing laboratories began routinely performing pooled testing to effectively extend the stock of remaining tests.

Here we aim to assess the efficiency of pooled testing for TB and to estimate the agreement between pooled and individual testing using routine data from pooled testing for these assays.

## Methods

### Setting and design

This was a retrospective analysis of pooled testing conducted using the Xpert MTB/RIF and Xpert MTB/RIF Ultra assays. Prior to December 2020, Cameroon used Xpert MTB/RIF (Xpert) assays for all TB testing on GeneXpert platforms; in December 2020, the country switched entirely to the Xpert MTB/RIF Ultra (Ultra) assays for TB testing. Pooled testing was conducted routinely at multiple reference and peripheral laboratories; data for this analysis were extracted from consecutive tests on samples pooled in pools of 2 or 3 conducted at two high-volume reference laboratories in Bamenda and in Douala. All methods were carried out in accordance with approved guidelines.

This evaluation was conducted together with the National TB Program. The study was approved by the Institutional Review Board of the Cameroon Baptist Convention Health Services (IRB2021-64), with waiver of informed consent. All testing data was de-identified prior to analysis.

### Clinical specimens

The laboratories receive specimens for diagnostic testing for both initial diagnosis of TB in people to be evaluated for TB and for detection of rifampin-resistant TB among people with confirmed TB. Pooling was conducted only on specimens for initial TB diagnosis. Only sputum specimens with a volume of at least 1 mL were included in pools; most specimens received had a sputum volume between 1 and 4 mL.

The decision to pool or to test individually was determined by the lab based on reagent availability and patient characteristics; specimens from patients with known smear-positive results or at higher risk of TB were mostly tested individually, while specimens from patients without these risk factors for TB were typically pooled. Typically, only one sputum specimen per person was tested using a rapid diagnostic, in line with national guidance.

### Pool size determination

Prior to starting pooled testing, the TB positivity rate was 7–12% at the different labs depending on site and population group. Based on this positivity range, the theoretical optimal pool size was 4^42,43^. The labs initially chose to use the more conservative pool size of 3 since pooling was not yet a widely adopted practice. In some cases, the labs used a pool size of 2 as determined by the lab based on current variables, including numbers of specimens available for pooled testing at the time the batch of specimens was to be tested.

### Sputum specimen processing and creation of pools

Sputum specimens to be pooled were initially processed following the manufacturer’s recommendation^44,45^. Briefly, a volume of the provided Xpert Sample Reagent (SR) of approximately two times the volume of the sputum was added to the sputum (for example, 3 mL of Xpert Sample Reagent was added to a sputum specimen of 1.5 mL). Then the sputum container was shaken and incubated at room temperature for 15 min, or for additional time until fully liquefied. Using this approach, each individual specimen-reagent mixture to be used for pooling had a volume of at least 3 mL. The Ultra reagents are provided with 8 mL of Xpert SR and one 3 mL disposable pipet per assay; for individual testing, one Xpert SR container per specimen is typically used. To perform pooled testing, additional disposable pipets are required, so that each specimen has one disposable pipet. The SR is added to the specimen using the disposable pipet (rather than by pouring) and the same Xpert SR container may then be used for multiple specimens, with the reagent pipetted and dispensed into the specimen using a new disposable pipet for each specimen.

To create the pool of specimens, approximately 0.9 mL of each individual specimen-reagent mixture was added to a dedicated sputum container for the pooled sample for pools of 3, and approximately 1.5mL of each mixture was added to create pools of 2. The leftover specimen-reagent mixture for each individual specimen was set aside; if the pool tested positive for MTB, this remaining sample-reagent mixture (with remaining volume of at least 2 mL) was used for individual testing of the specimen. The specimen-reagent mixture was stored and re-tested following the manufacturer’s guidance of within 4 h if stored at room temperature, or within 24 h if stored in the refrigerator at 2–8 °C.

### Xpert and Ultra testing- pooled and individual specimens

To perform the assay, slightly more than 2 mL of the pooled mixture was added to a single Xpert or Ultra cartridge, and the specimen was run on the GeneXpert instrument following the standard procedures for testing^44,45^. For specimen pools with a result of MTB NOT DETECTED, all specimens from the pool were then reported as MTB NOT DETECTED. For specimen pools with any result of MTB DETECTED, each specimen was re-run individually and each result was reported for each specimen individually. For invalid pools, either the pool or the individual samples were subsequently re-tested, depending on the error code and the amount of remaining specimen-reagent mixture for the pool.

### Data extraction and preparation

Xpert and Ultra testing data were extracted from the GeneXpert instrument. Pooled tests with positive results were matched to respective individual test results using the unique specimen numbers employed by the laboratories.

Assays with a result of MTB DETECTED (any grade) were classified as positive, assays with a result of MTB NOT DETECTED were classified as negative, and assays with a result of error, invalid or no result on the assay were classified as invalid. Only results of tests with positive or negative results were retained for analysis; the proportion of assays with invalid results was determined and used to estimate testing efficiency.

All analyzed data are included as supplemental material (Table S4).

### Statistical analysis

The number of overall pools and specimens tested, the number of pools and specimens that tested positive, and the total number of PCR tests performed were summarized, both overall and stratified by pool size and type of test.

The theoretical optimal efficiency for two-stage hierarchical testing, which assumes that positive specimens are equally distributed across pools and assay sensitivity and specificity are 100%, was determined as ${(\frac{n+1}{n}-{(1-p)}^{n})}^{-1}$, where n is the pool size and p is the prevalence of positive results^1^. The observed efficiency was determined by dividing the number of specimens with test results produced by the number of test cartridges used; the number of tests run per person with valid test result is the inverse. The percentage of cartridges saved due to pooled testing as compared to individual testing was estimated by dividing the number of tests used for pooled testing by the number of cartridges that would have been needed to test all specimens individually, including additional cartridges for anticipated invalid results.

The theoretical increase in cycle threshold from individual to pooled test is calculated as log_2_(n), where n is the pool size, as each PCR cycle approximately doubles the amount of targeted nucleic acid.

To evaluate the agreement between the cycle threshold (Ct) values for specimens that tested positive initially in a specimen pool and subsequently by individual testing, we used the Bland–Altman method^46,47^ and Passing-Bablok regression analysis^48^. Only specimens in pools of 3 with a single positive result of rifampin-sensitive TB were included.

To estimate the positive percent agreement between testing in pools of 3 as compared to individual testing, we conducted a retrospective in silico analysis similar to what has been reported to assess the performance of pooled testing with molecular diagnostics for SARS-CoV-2^20,49^. Passing-Bablok regression was used to determine the expected shift in cycle threshold for TB detection due to sample dilution when testing pools of three as compared to individual testing. Referring to historical data sets consisting of specimens tested individually, we then estimated the proportion of specimens in the comparison sets that would have been expected to have cycle threshold values in the detectable range after excluding those that would have had a cycle threshold value too high to be detected due to dilution from pooling. For each probe, we used the highest cycle threshold value in the data set as the highest value in the cycle threshold interval; this was chosen as a more conservative approach than using the assay cutoff cycle threshold for each probe.

All analyses were performed in R version 4.1.2; the method comparison analyses were performed using the ‘mcr’ package^50^.

## Results

**Figure 1:**
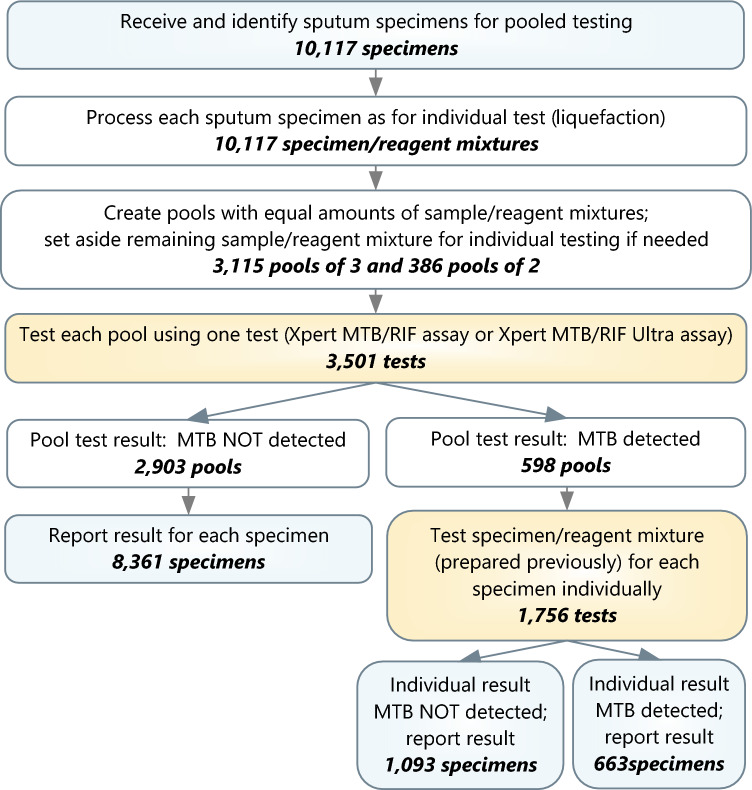
Flow of pooled testing for TB detection with the Xpert MTB/RIF and Xpert MTB/RIF Ultra assays at two reference laboratories (MTB: *Mycobacterium tuberculosis* complex; pools and individual tests with invalid results not shown). Overall, 10,117 specimens (blue) were tested in 3501 pools, with a 5257 Xpert or Ultra assays (orange), including 1756 assays run individually for each of the specimens in the 598 positive pools.

From June 1, 2020 to February 28, 2022, two reference laboratories tested 10,117 specimens in 3501 pools, including 3115 pools of 3 and 386 pools of 2, using the Xpert MTB/RIF and Xpert MTB/RIF Ultra assays, as shown in Fig. 1. Among the 598 pools with TB detected, after testing each specimen in these pools individually, there were 663 (6.6%) specimens with a result of TB detected and 1093 specimens with a result of TB not detected. All 8361 specimens in negative pools were reported as TB not detected.

### Testing efficiency

The efficiency of testing was determined for each assay and pool size from the total number of tested specimens divided by the number of cartridges used, as shown in Table 1, and compared to the theoretical efficiency for two-stage hierarchical pooling^1^. In total, 1590 pools were tested on the Xpert assay from June to December 2020, and 1911 pools were tested on the Ultra assay from December 2020 to February 2022. For pools of 3 on the Ultra assay, with 6.9% of specimens positive for TB, pooled testing in pools of 2 saved 40% of cartridges, while pooled testing in pools of 3 saved 49% of cartridges. Similar results were obtained with the Xpert assay, as shown in Table 1.

**Table 1:** Testing statistics and efficiency of specimen pooling using the Xpert MTB/RIF (Xpert) and Xpert MTB/RIF Ultra (Ultra) assays.

	Xpert		Ultra		Combined
	Pools of 2	Pools of 3	Pools of 2	Pools of 3	Total
Number of pools with results	107	1483	279	1632	3501
Number of pools with MTB detected	10	266	28	294	598
Number of specimens with test results produced	214	4449	558	4896	10,117
Number of positive specimens	12	285	28	338	663
Positivity rate	5.6%	6.4%	5.0%	6.9%	6.6%
Number of PCR tests (cartridges) used*	133	2385	350	2629	5498
Efficiency					
Theoretical Dorfman efficiency ${(n+1/n-{\left(1-p\right)}^{n})}^{-1}$; $n$ = pool size, $p$ = prevalence	1.64	1.95	1.67	1.90	
Observed empirical efficiency (# of specimens with results/ # of tests)	1.61	1.87	1.59	1.86	1.84
Number of tests run per person with result (# of tests/# of specimens with results)	0.62	0.54	0.63	0.54	0.54
Estimated additional number of people with test results (as compared to individual testing*)	71	1860	182	2043	4156
Estimated % of test cartridges saved (as compared to individual testing*)	41%	49%	40%	49%	48%

*Including 4.6% of assays (3.7% of Xpert and 5.3% of Ultra) with invalid results that were re-tested.

### Estimating the positive percent agreement between pooled and individual results (for pools of three)

To estimate the positive percent agreement between pooled and individual tests, we performed in silico analyses using the estimated shift in cycle threshold for pooled testing applied to the range of cycle threshold values from historical clinical data sets of individual test results.

**Figure 2:**
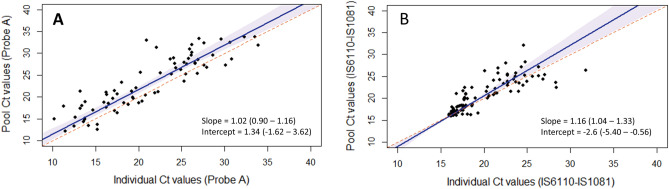
Passing-Bablok regression for pools of 3 for the Xpert MTB/RIF (**A**) and Xpert MTB/RIF Ultra (**B**) assays. For the Passing-Bablok regression, the dotted line is the line of equality, and the shaded area is the 95% confidence interval. (Only specimens in pools of three with only one positive and two negative results were included; 70 for Xpert and 119 for Ultra.)

To estimate the shift in cycle threshold values for the assays due to dilution from specimen pooling, we compared the cycle threshold values of specimens that tested positive on both pooled and individual testing, as shown in Fig. 2 (and in Table S2). For this comparison, we included only specimens in pools of 3 from Lab B that had one positive and two negative results.

For the Xpert MTB/RIF assay, 70 specimens were included in the analysis. The median cycle threshold value of the individual results for Probe A was 20.3 (interquartile range, 16.2 to 25.9), and the median cycle threshold value of the pooled results was 21.4 (interquartile range, 18.1 to 27.9).

For the Xpert MTB/RIF Ultra assay, 119 specimens were included. The median cycle threshold value of the individual results for the multicopy IS6110-IS1081 target was 17.6 (interquartile range, 16.4 to 21.6), and the median cycle threshold value of the pooled results was 18.1 (interquartile range, 16.4 to 22.8).

The comparison historical data set for the Xpert MTB/RIF assay were routine clinical specimens tested from January to May 2020; in this population, 6.5% (196/2,994) of those tested had a result positive for TB and 28% (54/196) of specimens with a result of TB detected had a grade of MTB detected very low, the lowest semi-quantitative result for this assay. For the Xpert MTB/RIF Ultra assay, the comparison historical data set was a population of individual specimens tested during a study that included 8 hospitals across the country; in this population, 15.1% (162/1068) of specimens had a result of TB detected. Of those with TB detected, 11.1% (18/162) had a result of trace, the lowest semi-quantitative result for this assay, and 12.3% (20/162) had a result of very low, the second lowest semi-quantitative result.

**Figure 3:**
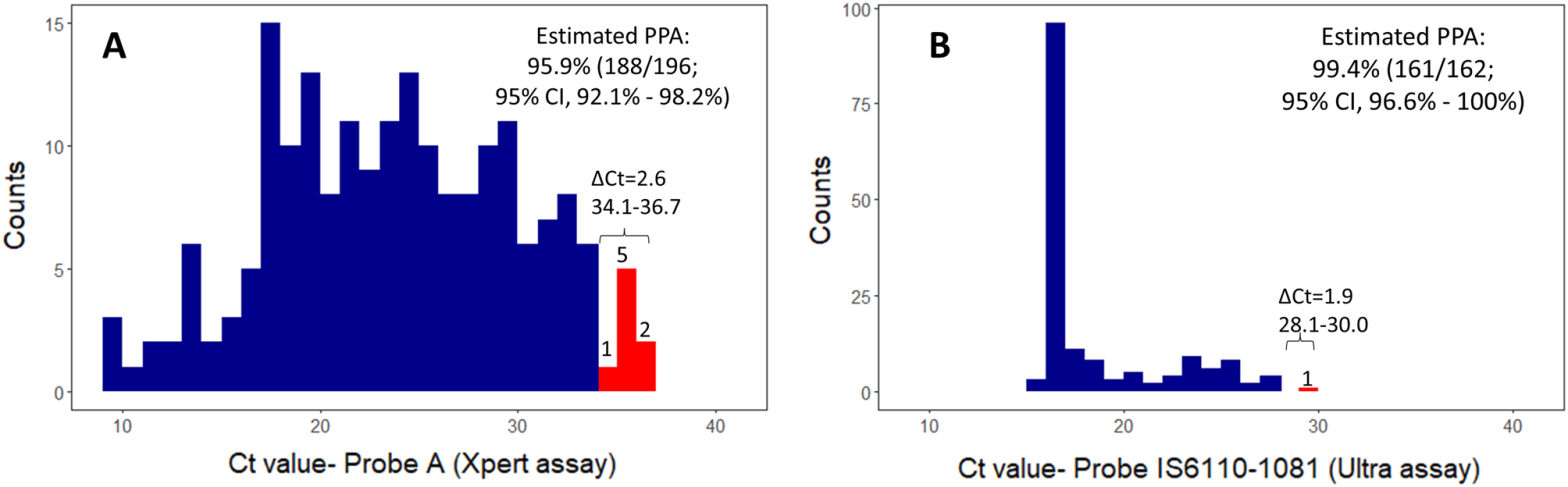
Estimated positive percent agreement (PPA) of pooled test results for pool size of three as compared to individual test results for the Xpert MTB/RIF and Xpert MTB/RIF Ultra assays based on in silico analysis, using historical data sets. (**A**) Histogram of historical clinical Xpert results with cycle threshold interval 34.1–36.7 obtained from Passing-Bablok regression (from Fig. 2A), as: *36.7* = *1.02x* + *1.34; with x* = *34.1;* 188 of 196 specimens (blue) would test positive on pooled testing with pools of 3, while 8 specimens (red) would test negative, giving a positive percent agreement of 95.9% (95%CI, 92.1–98.2%), as shown. (**B**) Histogram of historical clinical Ultra results, with cycle threshold interval 28.1–30.0 obtained from the Passing-Bablok regression (from Fig. 2B), as: *30.0* = *1.16x -2.6; with x* = *28.1*; 161 of 162 specimens (blue) would test positive on pooled testing with pools of 3 with 1 specimen (red) that would test negative, giving a positive percent agreement of 99.4% (95% CI, 96.6–100%), as shown.

The estimated positive percent agreement of the pooled results with individual results from the comparison data sets is shown in Fig. 3. For the Xpert assay, the cycle threshold shift for pools of 3 from individual to pooled testing was determined from Passing-Bablok regression as 2.6 cycles from a threshold value high of 36.7 (Fig. 3A, Table S2). There were 8/196 (4.1%) specimens from the comparison data set with cycle threshold values that fell in the interval between 34.1 and 36.7 cycles and would have been expected to be false negatives if tested in pools of 3. The estimated positive percent agreement between the pooled and individual results for the Xpert assay (with the target Probe A) is 95.9% (188/196, 95% CI, 92.1–98.2%). For the Xpert assay, the five probes used for TB detection (Probes A-E) each had between 2 and 8 specimens in the respective intervals (Table S3).

For the Ultra assay, the cycle threshold shift for pools of 3 from individual to pooled testing was determined from Passing-Bablok regression as 1.9 cycles from the threshold value high of 30 (Fig. 3B, Table S2). There was 1/162 (0.6%) specimen in the comparison historical data set with a cycle threshold value that fell in the interval from 28.1 and 30 cycles and would have been expected to be false negative if tested in pools of 3. The estimated positive percent agreement between pooled and individual test results for the Ultra assay, with the multi-copy target IS6110-IS1081 for *Mycobacterium tuberculosis* detection, is 99.4% (161/162, 95% CI, 96.6–100%).

### Discordant pool and individual test results

Among the Ultra pools of 3 from Lab B, there were 137 pools with TB detected, with 5 (3.6%) of these that were positive pools where none of the three specimens in the pool had an individual test result positive for MTB. These five pools had semi-quantitative results of MTB detected trace (with cycle threshold values of 25.4, 26.4, 26.5, 27.4, and 28.2), as shown in Figure S2. Among the Xpert pools of 3 analyzed, there were 83 pools with MTB detected, with 1 (1.2%) positive pool, with MTB detected low (with cycle threshold value of 22.6 cycles), that had all three individual results negative for TB.

## Discussion

Overall, 10,117 specimens were tested for TB using 5,498 rapid molecular tests. With pooled testing, we were able to test 4,156 additional specimens from people to be evaluated for TB than would have been possible with individual testing using the same number of test cartridges. With the Ultra assay, an estimated 0.6% (95% CI, 0–3.4%) of people with TB were missed on pooled testing with pools of 3 due to specimen dilution, and an additional approximately 3.6% (5 of 137 analyzed positive pools) were missed after failure to detect a positive result on individual testing after a pool tested positive for TB. Due to the limited availability of molecular assays, if pooled testing had not been adopted, many of these people would have instead only been able to access a smear microscopy test for TB, which has much lower sensitivity for TB detection (~ 50–60% for smear microscopy vs. 90% for Ultra, as compared to the reference standard of culture)^24,25^.

The testing efficiency was similar to the theoretical value, with an estimated 48% savings in test cartridges using pools of 3 in this population with a test positivity of 6.6%. We chose to use a pool size of 3 as a more conservative alternative than the optimal size of 4 for this prevalence (as determined using, for example, the app described here: https://www.chrisbilder.com/shiny/).^43^ Previous studies have reported that pooled testing using the Xpert and Ultra assays would have enabled similarly high proportions of cartridge savings^36,37,39^. During testing, 4.6% of assays run had an invalid result, similar to what has been reported elsewhere for these assays,^28^ which contributed to decreased testing efficiency as compared to the theoretical efficiency. The slighter lower than theoretical efficiency is different than some reports of pooled testing for SARS-CoV-2 where observed efficiencies were slightly better than theoretical efficiencies^16,51^. Higher than theoretical efficiency has been attributed to correlation effects when testing people in potential contact networks, for example, which improves the efficiency as compared to the assumption that all positive tests are evenly distributed across pools^51^.

Following the procedure described previously to estimate the positive percent agreement of pooled versus individual testing for RT-PCR assays using an in silico analysis^20,49^, we found that, for the pools of 3 used here, the estimated positive percent agreement was approximately 96% for the Xpert assay and 99% for the Ultra assay. The higher agreement for pooled testing with pools of 3 on the Ultra assay is likely attributable to the mechanism of detection of TB for this assay. In the Xpert MTB/RIF assay, TB detection is based on the amplification of portions of the *rpoB* gene, while for the Xpert MTB/RIF Ultra assay, TB detection is based on amplification of portions of the multi-copy IS1081 and IS6110 insertion elements, with reported limits of detection of approximately 113CFU/mL, and 16CFU/mL for each assay respectively^52^. The histogram of cycle threshold values for the Ultra assay is skewed to lower values as compared to the histogram of the values for the Xpert assay (as in Fig. 2), since detection of the multi-copy insertion elements generally requires fewer cycles of amplification. For the in silico analysis, we used the cycle threshold shift determined from Passing-Bablok regression; using either the difference in median cycle threshold values between pooled and individual tests or the bias from the Bland–Altman analysis would lead to similar estimates of the positive percent agreement. The positive percent agreement estimated using this approach was also similar to that reported in studies where all specimens were tested both individually and in pools on the Xpert or Ultra assays^36,37,39^.

Among the analyzed positive pools of three on the Ultra assay, 3.6% (5/137) had an initial pooled test result of MTB detected while the three individual specimens had results of MTB not detected. Similar rates of positive pool results followed by all individual specimens in the pool testing negative were reported by Barak et al. (3.9–5.3%)^16^ and Wang et al. (2.5–2.9%)^53^ for SARS-CoV-2 PCR testing. There are several possible factors that may contribute to these discordant results. The manufacturer evaluated the reproducibility of Ultra assay results for a sample with a low concentration of rifampin-sensitive MTB; IS1660/IS1081 targets for the 144 tests on the same standard simulated specimen had a mean cycle threshold of 23.7, with a standard deviation of 1.80 cycles (coefficient of variation of 7.6%). Nearly all of the variance was due to the assay (standard deviation of 1.7), rather than due to the lot, the day, the site or the operator^45^. Variance in assay performance is attributed to variations in chemical efficiencies (of the enzymes, primer and/or template) and variable equipment factors including fluorescence detection, temperature and variations in reagent volume^54,55^. In this work, positive pool results followed by negative results for all individual specimens in the pool occurred for 5 pools with results of MTB trace, with cycle threshold values of 26.4–28.2 (Figure S2). Pools with weakly positive results followed by subsequent individual negative results may be due to assay reproducibility issues; when the sample/reagent mixture was tested individually after a positive pool result, the assay did not reproducibly detect the very low quantity of *Mycobacterium tuberculosis* complex that was present in the specimen on the second assay. This interpretation is supported by the variation in the cycle threshold values at higher values, as in Figure S2. Another source of variability in results between pooled and individual testing may be degradation of the target DNA between tests^55^; however, since we performed individual testing on specimens from positive pools within 4 h if stored at room temperature and within 24 h if stored at 2–8 °C, as recommended by the manufacturer, we did not consider this to be a likely contributor to discordant results. In these rare cases of discordant pool and individual results, either a second submitted specimen or new specimens from each person to be evaluated for TB with a specimen in the pool can be collected and tested on Ultra for TB detection, and/or these individuals can also be followed up clinically for a diagnostic decision.

The use of the Xpert MTB/RIF Ultra assay, with the additional trace positive result category, enables more sensitive detection of TB than possible with the Xpert MTB/RIF assay^25,56,57^. However, molecular testing for TB, even with the Ultra assay, does not detect TB in all people with TB disease, and clinical follow-up and diagnosis is an important component of TB care even with individual (non-pooled) molecular testing^22^. It may be preferable to perform individual testing in some populations that are more likely to have paucibacillary TB with MTB trace results, such as hospitalized people living with HIV and people living with HIV not receiving ART, due to their higher risk of dying due to having both HIV and TB and the benefit of using the most sensitive method possible to aid in TB diagnosis^21^. Among other populations that are more likely to have paucibacillary TB, such as people evaluated for TB earlier in the course of the disease during active case finding, pooled testing may be preferred to individual testing because many more people could be tested as compared to individual testing, even if some people with TB trace results may be missed. Further evaluations of pooled testing performance, efficiency and cost effectiveness in different populations will provide more evidence that can be used to inform future guidance in this area.

With the approach described here, pooled testing and subsequent individual re-testing for samples from positive pools are performed on the same sputum specimen. In rare cases, a second sputum specimen is required if the test fails and there is insufficient sputum left for re-testing of the first specimen, or if the pooled result is positive and all individual results are negative (in this work 3.6% of positive pools). For individual testing, it is also necessary to have a second specimen when the first specimen produces an invalid result. In some programs and laboratories, two specimens are collected routinely, which facilitates immediate re-testing in these scenarios; in others, a second sputum specimen is requested from the client only in the case of a failed test or for other follow-on testing. The WHO recommends that programs may consider collecting two sputum specimens routinely for such operational reasons^22^.

This work had several strengths. As it was a retrospective evaluation, the efficiency and performance analyses reflect those encountered under routine conditions, and results from more than 10,000 specimens were included. There were also limitations. Due to the specimen identification tracking procedure in one of the labs, it was not possible to unambiguously determine whether individual tests for a particular person after a positive pooled test result was from the first specimen or from a second specimen from the same person. For this reason, data from this lab were included in the summary statistics (Table 1) but were not included in other analyses. However, we were able to include 70 Xpert results and 119 Ultra results in the method comparison analyses, which provided robust estimates. For the in silico analyses, we included historical clinical data sets with 196 (Xpert) and 162 (Ultra) positive specimens; including data sets with more specimens would enable better estimates of the positive percent agreement. Because this was work conducted under routine testing conditions, data on individual participant characteristics were not collected and analyzed. Since specimens from people at higher risk of rifampin-resistant TB were tested individually, there were few pool results with rifampin resistance, and these were not analyzed here. Also, we did not evaluate the overall cost-effectiveness of this intervention, though we report that 48% of cartridges were spared by using pooled testing; due to these savings in cartridges, the cartridge cost per specimen was approximately $5.19, as compared to the full individual Ultra cartridge cost of $9.98 (at the current price in eligible countries)^58^.

## Conclusions

These results suggest that in this population, of 1000 people where 69 people have tuberculosis detectable with an individual Ultra test, pooled testing using a pool size of three with the Ultra assay will miss approximately 1 case due to sample dilution and 2 cases due to test reproducibility issues in performing the individual test after the sample pool tests positive (although some of these cases might be detected by testing a second specimen or by clinical diagnosis). With pooled testing in pools of 3, these 1000 people could be tested using only 543 cartridges, and an additional 840 people could be tested with the remaining 457 cartridges, resulting in the potential detection of an additional 56 people with TB.

These results support the use of pooled testing to improve the efficiency of TB detection in resource-constrained situations.

### Supplementary Information

Supplementary Information 1.

Supplementary Information 2.

## Data Availability

All analyzed data are included in the Supplementary Information files.
