# Supplementary material for: Implementation of large-scale pooled testing to increase rapid molecular diagnostic test coverage for tuberculosis: a retrospective evaluation
Source: Sci Rep. 2023 Sep 16;13:15358. doi: 10.1038/s41598-023-41904-w (PMC10505184; doi:10.1038/s41598-023-41904-w)
Supplement: Supplementary file 1 — Supplementary Information 1. [file 41598_2023_41904_MOESM1_ESM.pdf]

Supplementary Information.

Implementation of large-scale pooled testing to increase rapid molecular diagnostic test coverage for tuberculosis: a retrospective evaluation

Comfort Vuchas, Pride Teyim, Beh Frankline Dang, Angela Neh, Liliane Keugni, Mercy Che, Pantilius Nji, Hamada Beloko, Victor Fondoh, Norah Nyah Ndi, Irene Adeline Goupeyou Wandji, Mercy Fundoh, Henri Manga, Cyrille Mbuli, Jacob Creswell, Annie Bisso, Valerie Donkeng, Melissa Sander

Table S1. Pooled testing statistics and summary of test results for pools of 3 tested on the Xpert MTB/RIF and Xpert MTB/RIF Ultra assays, by testing lab, from June 2020 to Feb 2022

	Xpert MTB/RIF			Xpert MTB/RIF Ultra		
	**Lab A**	**Lab B**	**Total**	**Lab A**	**Lab B**	**Total**
Number of specimens tested	2,613	1,836	4,449	1,788	3,108	4,896
Total number of pools	871	612	1,483	596	1,036	1,632
Number pools with MTB detected	183	83	266	157	137	294
Pool results, semi-quantitative assay result						
MTB detected, trace	-	-	-	25	26	51
MTB detected, very low	30	19	49	18	24	42
MTB detected, low	43	21	64	52	42	94
MTB detected, medium	75	30	105	37	21	58
MTB detected, high	35	13	48	25	24	49
Number individual specimens with MTB detected	195	90	285	190	148	338
Individual results, semi-quantitative assay result						
MTB detected, trace	-	-	-	23	24	47
MTB detected, very low	31	8	39	29	22	51
MTB detected, low	46	31	77	59	51	110
MTB detected, medium	66	27	93	33	18	51
MTB detected, high	52	24	76	46	33	79
Number of positive specimens in pool with 1 positive in pool	141	74	215	117	119	236
Number of positive specimens in pool with >1 positives in pool	54	16	70	73	26	99

Of the 1,483 pools of 3 tested on the Xpert MTB/RIF assay, 871 were tested at Lab A, with 612 tested at Lab B; 7.5% (195/2,613) of specimen results were positive for MTB at Lab A, and 4.9% (90/1,836) were positive for MTB at Lab B (as shown in Table S1). Of the 1,632 pools of 3 tested on the Xpert MTB/RIF Ultra assay, 596 were tested at Lab A, with 1,086 tested at Lab B; 10.6% (190/1,788) of specimen results were positive for MTB at Lab B, and 4.8% (148/3,108) were positive at Lab B.

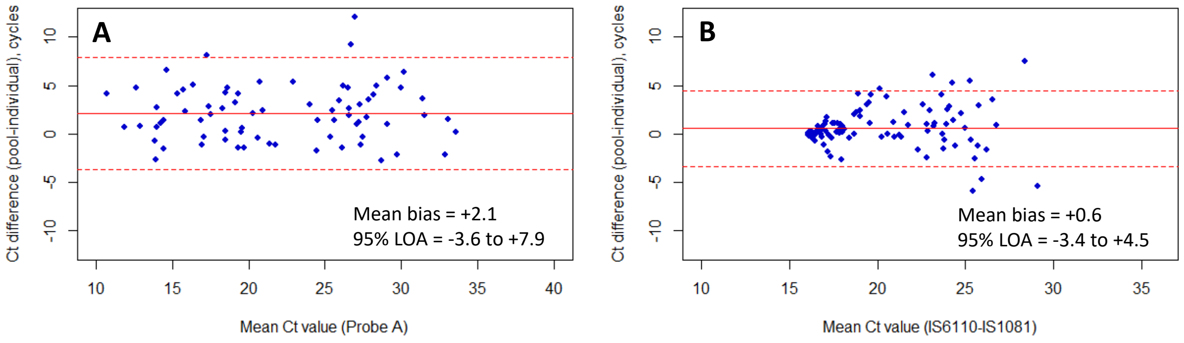

Figure S1. Bland-Altman plots for pools of 3 tested the Xpert MTB/RIF and Xpert MTB/RIF Ultra assays. The solid red line indicates the mean bias and the dashed red lines indicate the 95% limits of agreement (LOA). Only specimens in pools of three with one positive and two negative results were included; 70 were included for Xpert and 119 for Ultra.

For the Xpert MTB/RIF assay Probe A, the mean bias between the pooled and individual cycle threshold values was +2.1 cycles (95% limits of agreement, -3.6 to +7.9). For the Xpert MTB/RIF Ultra Probe IS6110-1081, the mean bias between the pooled and individual threshold values was +0.6 cycles (95% limits of agreement, -3.4 to +4.5).

Table S2. Estimation of cycle threshold (C_t) value shifts from individual to pooled testing for pools of 3. Based on Passing Bablok regression, for Xpert and Ultra probes analyzed for specimens that tested positive on both pooled and subsequent individual testing; the highest cycle threshold value for each target was taken from historical data sets (as shown in histograms in Figure 3)

Assay	Target description	Number of positive specimens in group, with individual and pool Ct results	From Passing-Bablok regression (Figure 2) Slope (95% CI)	Intercept (95% CI)	ΔCt calculated for pooled vs. individual testing, (Passing-Bablok regression)	Highest Ct for MTB detection on individual specimen*	Estimated highest Ct of individual specimen for MTB detection in pool of 3, due to specimen dilution
Xpert MTB/RIF (G4 v.6)	ProbeA (rpoB)	70	1.05 (0.93 to 1.19)	0.88 (-1.81 to 3.44)	2.59	36.7	34.1
Xpert MTB/RIF (G4 v.6)	ProbeB (rpoB)	70	1.01 (0.90 to 1.12)	1.37 (-1.34 to 4.14)	1.72	37.0	35.3
Xpert MTB/RIF (G4 v.6)	ProbeC (rpoB)	70	1.02 (0.90 to 1.14)	1.15 (-1.45 to 3.98)	1.86	37.2	35.3
Xpert MTB/RIF (G4 v.6)	ProbeD (rpoB)	70	1.03 (0.91 to 1.16)	1.19 (-1.58 to 3.67)	2.28	38.5	36.2
Xpert MTB/RIF (G4 v.6)	ProbeE (rpoB)	70	1.08 (0.93 to 1.22)	0.45 (-2.53 to 3.66)	3.34	39.4	36.1
Xpert MTB/RIF Ultra (v.4)	IS6110-IS1081 (all Ct values)	119	1.16 (1.04 to 1.33)	-2.6 (-5.40 to -0.56)	1.90	30	28.1
	IS6110-IS1081 (Ct >16.5)	74	1.04 (0.87 to 1.22)	-0.11 (-3.5 to 3.3)	1.05	30	29.0
	IS6110-IS1081 (Ct >18)	46	0.96 (0.66 to 1.46)	1.62 (-8.75 to 8.41)	0.44	30	29.6

Table S3. Estimated positive percent agreement (PPA) of pooled test results for pool size 3 as compared to individual test results for the Xpert MTB/RIF and Xpert MTB/RIF Ultra assays; based on in silico analysis, using two comparison data sets (as shown in histograms in Figure 3)

Assay (Target)	ΔCt calculated (based on Passing-Bablok regression)	Estimated Ct interval in which positive specimens in pools expected to test negative due to sample dilution from pooling*	Clinical specimens tested individually, as comparison population		Proportion of specimens with Ct in interval		**Estimated positive percent agreement between pooled and individual test result (PPA, 95% CI)**
			Number with MTB detected	Cycle threshold median (IQR)			
Xpert MTB/RIF G4 v.6 (Probe A)	2.59	[34.1, 36.7]	196	23.5 (19.2 - 28.8)	4.1%	8	**95.9% (92.1% to 98.2%)**
Xpert MTB/RIF G4 v.6 (Probe B)	1.72	[35.3, 37]	196	25.0 (20.7 - 29.1)	1.0%	2	
Xpert MTB/RIF G4 v.6 (Probe C)	1.86	[35.3, 37.2]	196	24.0 (19.5 - 28.8)	1.5%	3	
Xpert MTB/RIF G4 v.6 (Probe D)	2.29	[36.2, 38.5]	193	24.7 (20.4 - 29.6)	2.6%	5	
Xpert MTB/RIF G4 v.6 (Probe E)	3.34	[36.1, 39.4]	190	24.8 (20.6 - 30.4)	3.7%	7	
Xpert MTB/RIF Ultra (IS6110-IS1081)	1.90	[28.1, 30]	162	16.5 (16.3-20.5)		0.6% (1/162)	**99.4% (96.6% to 100%)**

Figure S2. Comparison of pooled and individual cycle threshold (Ct) values for pools of 3 on the Xpert MTB/RIF Ultra assay (with the IS6110-IS1081 target). Paired Ct values for pools of 3 with 1 positive and 2 negative specimens per pool, for 124 specimens.

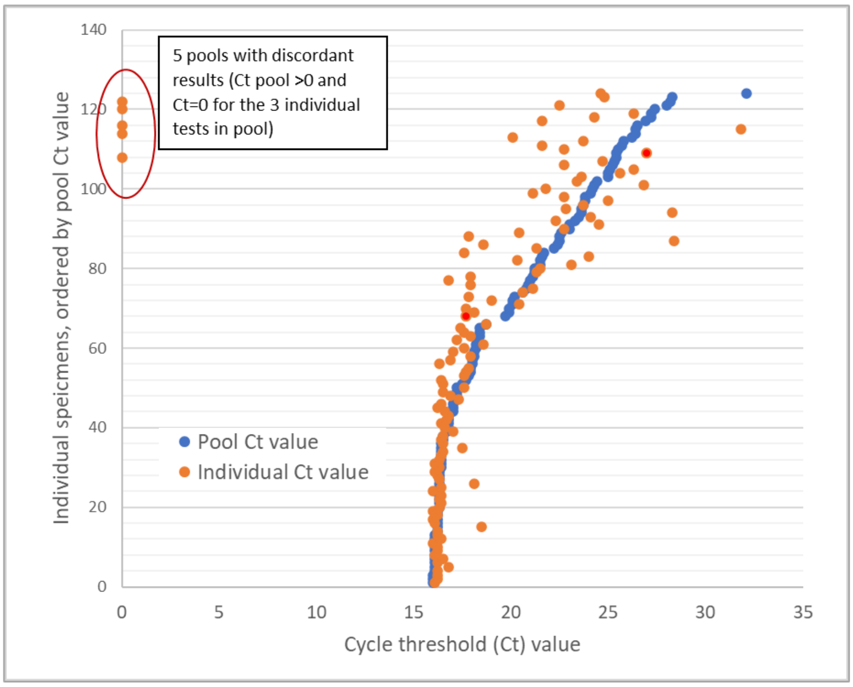

Table S4. Raw data of control and bacterial Ct values of analysed pools and individual specimens, by assay (Xpert or Ultra).

(See separate csv file)
